# Profilin 2 promotes growth, metastasis, and angiogenesis of small cell lung cancer through cancer-derived exosomes

**DOI:** 10.18632/aging.202213

**Published:** 2020-11-21

**Authors:** Qi Cao, Yihan Liu, Ying Wu, Caijiao Hu, Lei Sun, Jinghui Wang, Changlong Li, Meng Guo, Xin Liu, Jianyi Lv, Xueyun Huo, Junming Yue, Xiaoyan Du, Zhenwen Chen

**Affiliations:** 1 School of Basic Medical Sciences, Capital Medical University, Beijing Key Laboratory of Cancer Invasion and Metastasis Research, Beijing 100069, China; 2 Department of Pathology, Shandong Chest Hospital, Shandong 250020, Jinan, China; 3 Department of Medical Oncology, Beijing Chest Hospital, Capital Medical University, Beijing Tuberculosis and Thoracic Tumor Research Institute, Beijing 101149, China; 4 Center for Cancer Research, School of Medicine, The University of Tennessee Health Science Center, Memphis, TN 38163, USA

**Keywords:** profilin 2, small cell lung cancer, metastasis, angiogenesis, exosome

## Abstract

Small cell lung cancer (SCLC) is highly aggressive and prone to hypervascular metastases. Recently, we found profilin 2 (PFN2) expression in SCLC but not in normal tissues. Furthermore, PFN2 expression had been shown to promote angiogenesis through exosomes. However, it remains unclear whether PFN2 contributes to the progression and metastasis of SCLC through angiogenesis. We report here that overexpression (OE) of PFN2 increased, whereas its knockdown (KD) decreased the proliferation, migration, and invasion of SCLC cell H446. The exosomes from OE-H446 (SCLC-OE-exo) exhibited similar effects on H446 properties. Culturing of endothelial cells (ECs) in SCLC-OE conditioned medium (CM) or SCLC-OE-exo increased the migration and tube formation ability of ECs, whereas SCLC-KD-CM and SCLC-KD-exo had inhibitory effects. Interestingly, both SCLC- and EC-derived exosomes were internalized in H446 more rapidly than in ECs. More importantly, OE-PFN2 dramatically elevated SCLC growth and vasculature formation as well as lung metastasis in tumor xenograft models. Finally, we found that PFN2 activated Smad2/3 in H446 and pERK in ECs, respectively. Taken together, our study revealed the role of PFN2 in SCLC development and metastasis, as well as tumor angiogenesis through exosomes, providing a new molecular target for SCLC treatment.

## INTRODUCTION

Small cell lung cancer (SCLC), a fatal disease that accounts for 10%–15% of all lung cancer cases, is a subtype of lung cancer characterized by high aggressiveness and metastasis [1, 2]. Due to the rapid growth, early metastasis, and drug resistance of SCLC, the 5-year survival rate in patients with SCLC is <5%–7% [3, 4]. Furthermore, there has been a lack of therapeutic progress during the past decades. The precise pathogenesis of SCLC also remains unclear, and currently, there are no effective biomarkers for its early diagnosis and prognosis [1]. The SCLC is prone to hypervascular metastases, suggesting that a therapeutic approach targeting angiogenesis would be a good treatment strategy [5–7]. However, the molecular mechanisms underlying angiogenesis in SCLC have not been well-investigated. Thus, it is essential to elucidate angiogenesis signaling in the growth and metastasis of SCLC to improve the clinical treatment of SCLC.

Profilins are actin-binding proteins involved in the regulation of cytoskeletal dynamics. There are four members in the profilin family, namely, profilin 1, 2, 3, and 4 [8]. Several studies have shown that profilin 2 (PFN2) is specifically expressed in neuronal cells and kidney cells [9]. PFN1 and PFN2 have distinct roles in regulating synaptic actin polymerization in mouse brains, and PFN2 acts preferentially through a WAVE-complex-mediated pathway [10]. Recent evidence has revealed that PFN2 is a potential therapeutic target and biomarker for prognosis in esophageal squamous, head and neck squamous carcinomas [11, 12]. PFN2 has also been investigated in colorectal cancer cells [13] and in ovarian cancer [14]. Previous studies have shown that PFN2 contributes to epithelial-mesenchymal transition (EMT) and regulates Smad2/3 expression in non–small-cell lung cancer [15, 16]. However, it is unknown whether PFN2 plays a role in SCLC. Our primary study showed that PFN2 significantly promoted the proliferation, migration, and tube formation ability of HUVEC cells, and enriched presence of PFN2 in exosomes derived from endothelial cells (ECs) exerted the same influence on these cells (data not published). Therefore, to characterize the angiogenesis of SCLC, we examined PFN2 expression level in SCLC tissues. PFN2 expression was significantly higher in tissues obtained from SCLC patients than in normal tissues. Based on these results, we further investigated whether PFN2 promotes angiogenesis and eventually facilitates the growth and metastasis of SCLC.

Because PFN2 is not a secretory protein but a cytoskeleton regulatory protein, we hypothesized that similarly to ECs, SCLC cells release exosomes containing PFN2 to facilitate the cross-talk between SCLC and EC. Exosomes are extracellular vesicles (sized 50–100 nm) that mediate the cross-talk between tumor cells and stroma cells, such as vascular ECs and fibroblasts [17]. Accumulating studies have shown that cancer cell-derived exosomes induce vascular formation and permeability, promote angiogenesis, and thus facilitates tumor growth and metastasis [18, 19]. Exosomes with the various cargo are potentially valuable markers in the diagnosis and treatment of lung cancer [20]; however, their function, particularly in the growth and metastasis of SCLC, has not been well studied. Here, we aimed to evaluate the role of PFN2 in the growth, metastasis and angiogenesis of SCLC mediated by exosomes, to explore if PFN2 is a potential new therapeutic target for SCLC treatment.

## RESULTS

### PFN2 expression is upregulated in patients with SCLC

**Figure 1:**
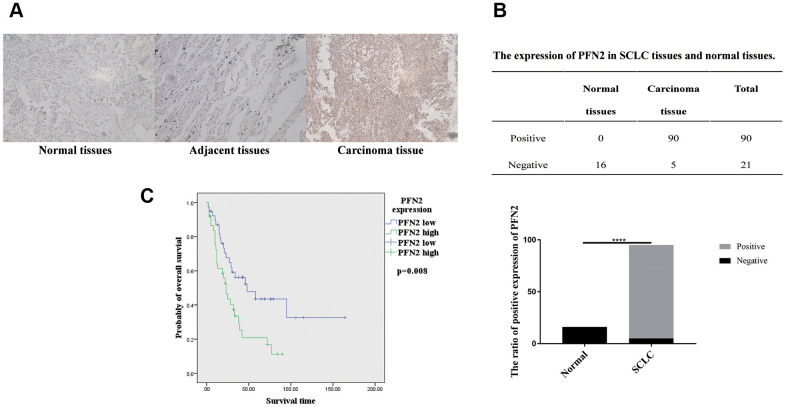
**PFN2 detected in SCLC tissue and normal lung tissue.** IHC results showing PFN2 expression in SCLC tissues but not in the healthy lung and adjacent tissues (**A**). The ratio of PFN2 expression is dramatically higher in SCLC tissue than in normal lung tissue (*P*=0.0000) (**B**). Kaplan-Meier survival analysis reveals that PFN2 expression is inversely correlated with the survival time in patients with SCLC (**C**).

First, we searched the TCGA database and the human protein atlas (https://www.proteinatlas.org/) to examine PFN2 expression in SCLC tissues and found that there were no data available on SCLC. However, data from the TCGA database and the human protein atlas showed that PFN2 is highly expressed in non–small cell lung cancer (NSCLC) but not in normal lung tissues (Supplementary Figure 1). Next, we collected 95 samples of SCLC tissue from patients pathologically diagnosed with SCLC, detected PFN2 expression using immunohistochemistry (IHC), and compared the expression with that in 6 adjacent normal tissues and 10 lung tissues from healthy individuals. Consistent with its expression in NSCLC, PFN2 was highly expressed in SCLC tissues, compared to adjacent normal and healthy lung tissues (Figure 1A, 1B). The ratio of positive PFN2 expression in SCLC and control groups was 94.7% vs. 0%, respectively (p=0.000, p<0.01). Kaplan-Meier survival analysis of TCGA data revealed that PFN2 expression was inversely correlated with the survival time in patients with SCLC (Figure 1C).

### PFN2 promotes proliferation, migration, and invasion of SCLC cells

**Figure 2:**
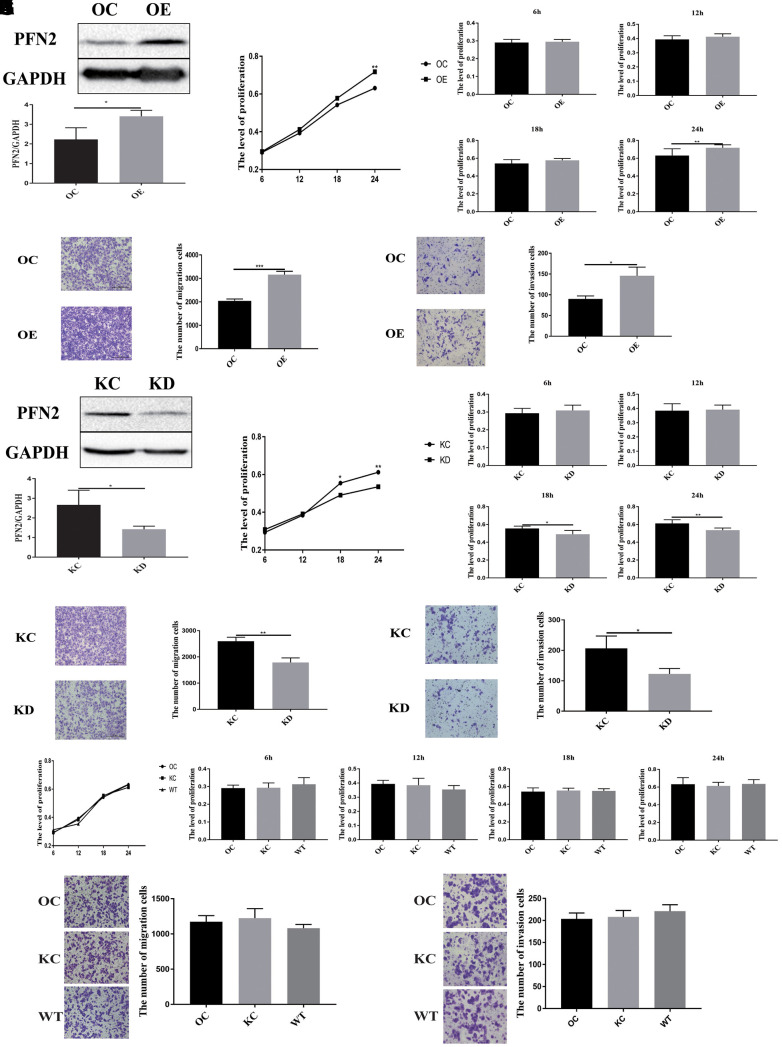
**PFN2 overexpression promotes the proliferation, migration, and invasion of SCLC cells.** PFN2 expression is significantly increased in H446 cells with PFN2 overexpression (*P*=0.02) (**A**). PFN2 overexpression shows significantly improved proliferation (*P*=0.6133, 0.1860, 0.0956, and 0.0278 at 6 h, 12 h, 18 h, and 24 h, respectively) (**B**), migration (*P*=0.0001) (**C**), and invasion (*P*=0.018) (**D**) of H446 cells. PFN2 expression is significantly decreased in H446 cells with PFN2 knockdown (*P*=0.03) (**E**). PFN2 knockdown shows markedly inhibited proliferation (*P*=0.3740, 0.7718, 0.0114, and 0.0027 at 6 h, 12 h, 18 h, and 24 h, respectively) (**F**), migration (*P*=0.002) (**G**), and invasion (*P*=0.0301) (**H**) of H446 cells (n=3). Overexpression control (OC), knockdown control (KC), and wild type (WT) of H446 cells show similar proliferation (*P*=0.1208, 0.2105, 0.7942, and 0.03275 at 6 h, 12 h, 18 h, and 24 h respectively) (**I**), migration (*P*=0.2721) (**J**), and invasion (*P*=0.3603) (**K**).

To explore whether PFN2 plays a role in SCLC tumorigenicity and metastasis, we first established the H446 cell line stably overexpressing PFN2 (referred to as H446-OE) compared to control cells (Figure 2A). We then examined the effect of PFN2 on cellular biological functions. The MTT and transwell assays indicated that PFN2 overexpression (OE) significantly increased proliferation, migration and invasion in H446 cells (Figure 2B–2D). Meanwhile, PFN2 knockdown in H446 cells (referred to as H446-KD) was also established, in which PFN2 was significantly reduced compared to that in the control line (Figure 2E). PFN2 knockdown significantly inhibited proliferation, migration, and invasion in H446 cells (Figure 2F–2H). Comparison tests showed that the OE control (H446-OC), knockdown control (H446-KC), and the wild type (H446-WT) of H446 cells had similar results (Figure 2I–2K), implying no influence of the respective modifications of H446 cells on their proliferation, migration, and invasion abilities. These results indicate that PFN2 significantly promotes the growth and invasion of SCLC cells.

### PFN2 functions in SCLC via exosomes

**Figure 3:**
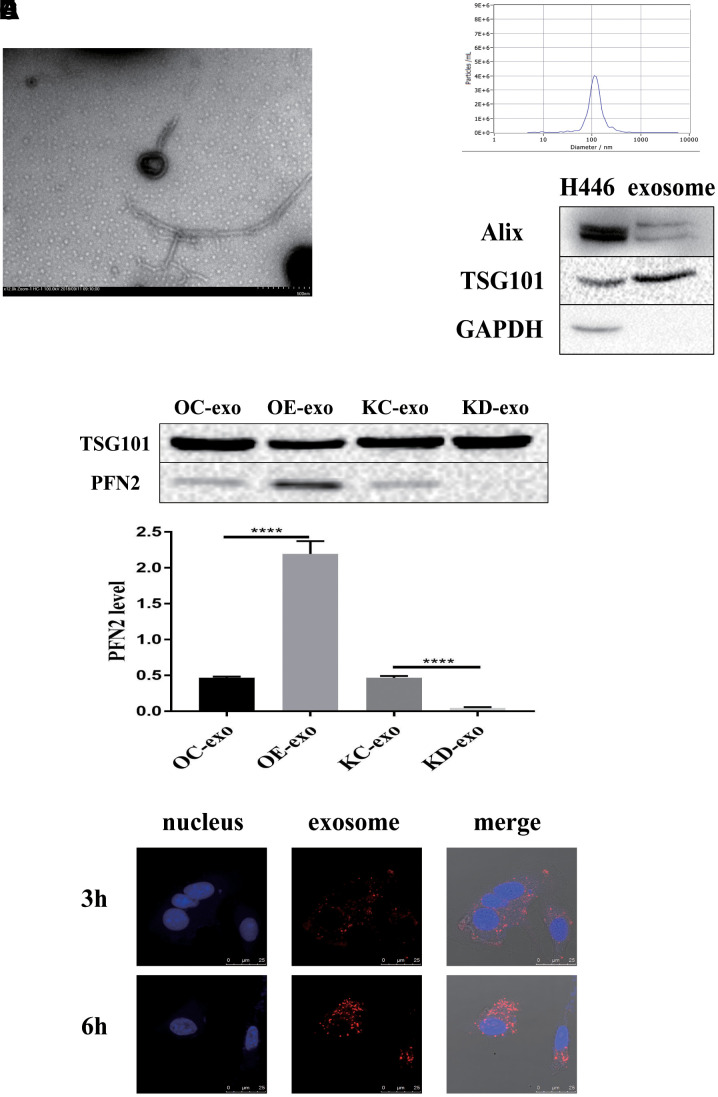
**Exosomes derived from H446 cells.** The exosomes are identified using TEM, NTA and western blot (**A–C**). PFN2 expression in exosomes derived from different kinds of H446 is detected using western blot (**D**). Exosomes can be internalized by H446 cells (**E**).

**Figure 4:**
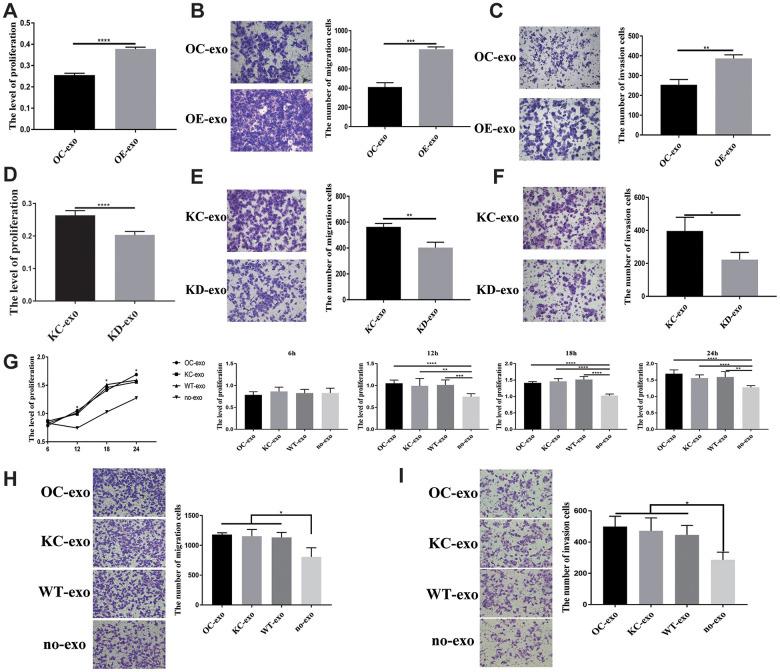
**Exosome derived from SCLC showing influence on H446 cells.** OE-exo promoted the proliferation (*P*=0.000) (**A**), migration (*P*=0.0002) (**B**), invasion (*P*=0.0019) (**C**) of H446 cells. Conversely, KD-exo inhibited proliferation (p=0.000) (**D**), migration (p=0.0051) (**E**), invasion (*P*=0.03) (**F**) of H446 cells. Exosomes from H446-OC, H446-KC, and H446-WT have similar function in promoting the proliferation (*P*=0.5288, 0.0076, 0.000 and 0.0014 at 6 h, 12 h, 18 h, and 24 h, respectively) (**G**), migration (*P*=0.0350) (**H**), and invasion (*P*=0.0231) (**I**) of H446 cells, which could significantly promote the proliferation, migration and invasion of H446 cells compared with that of control cells non-treated with exosomes (no-exo).

Considering that PFN2 is a cytoskeleton regulatory protein, we hypothesized that PFN2 could be loaded on exosomes. To test this, we collected the culture medium of H446-OE and H446-KD and isolated exosomes by ultracentrifugation. The presence of exosomes was confirmed by electron microscopic detection (Figure 3A), nano-transforming analysis (Figure 3B), and western blotting (Figure 3C). As shown in Figure 3A, 3B, the average size and diameter of the exosomes secreted by H446 were 100 nm, and most diameters ranged from 80 nm to 110 nm, which was consistent with findings of previous report [21]. Western blot analysis showed that the exosome markers of Alix and TGS101 were expressed (Figure 3C). Meanwhile, the exosomes from H446-OE (H446-OE-exo) harbored significantly higher PFN2 levels, whereas those from H446-KD (H446-KD-exo) displayed lower PFN2 expression than those in their corresponding control (Figure 3D). We labeled (PKH26, red) H446-derived exosomes (referred to as H446-exo) to test whether exosomes could be internalized by H446 cells. As expected, H446-exo was internalized by H446 cells within 3 h (Figure 3E). Lastly, we tested the impact of H446-OE-exo and H446-KD-exo on SCLC cell biological function. We found that compared with the corresponding control exosomes, H446-OE-exo dramatically increased the proliferation, migration, and invasion of SCLC cells (Figure 4A–4C), whereas H446-KD-exo significantly inhibited the proliferation, migration, and invasion (Figure 4D–4F). Moreover, we found that exosomes from H446-OC, H446-KC, and H446-WT had similar function in promoting the proliferation, migration, and invasion abilities of H446 cells, which were significantly enhanced compared with those in the group of control cells non-treated with exosomes (no-exo) (Figure 4G–4I). All these data strongly demonstrate that exosomal PFN2 significantly participates in SCLC biological functions.

### PFN2 from SCLC cells promotes migration and tube formation ability of HUVEC cells through exosomes

**Figure 5:**
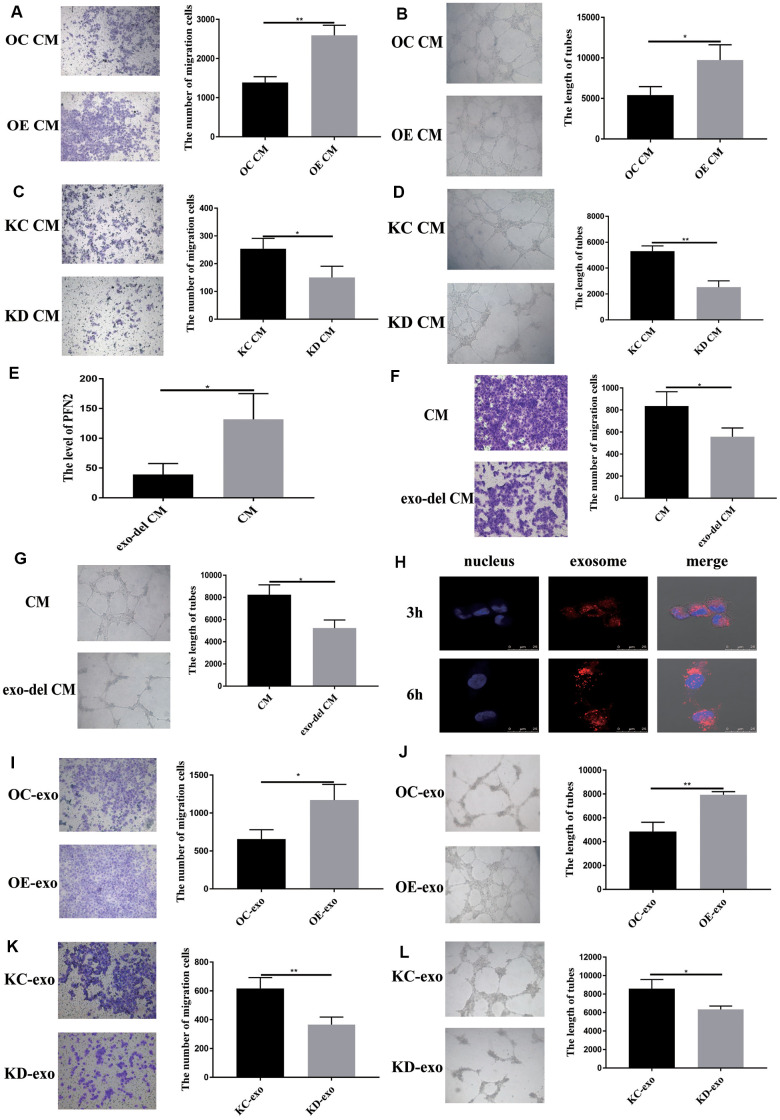
**Exosomal PFN2 from H446 cells affect migration and tube formation ability of HUVEC cells.** Co-culturing with H446-OE conditioned medium promoted (**A**) migration (*P*=0.0022) and (**B**) tube formation ability (*P*=0.0255) of HUVEC cells. Co-culturing with H446-KD cells conditioned medium remarkably decreased the migration (*P*=0.0319) (**C**) and tube formation ability (*P*=0.0017) (**D**) of HUVEC cells. When the exosomes are removed from the CM, PFN2 expression in the CM is significantly decreased (*P*=0.0267) (**E**). Exosome depletion from the CM weakens the migration (*P*=0.035) (**F**) and tube formation ability (*P*=0.0112) (**G**) of HUVEC cells. Exosomes derived from H446 cells could be internalized in HUVEC cells (**H**). OE-exo elevated the migration (*P*=0.02) (**I**) and tube length (*P*=0.0029) (**J**) of HUVEC cells. However, KD-exo inhibited the migration (*P*=0.0093) (**K**) and tube length (*P*=0.03) (**L**) of HUVEC cells.

To examine whether exosomes from SCLC affect EC angiogenesis, we cultured HUVEC cells in H446-OE or H446-KD conditioned medium (CM) or exosome-depleted CM. The results demonstrated that H446-OE-CM significantly increased the migration and tube length of HUVEC cells compared to H446-OC-CM (Figure 5A, 5B), whereas H446-KD-CM significantly inhibited the migration and tube length of HUVEC cells compared to H446-KC-CM (Figure 5C, 5D). We removed the exosomes from the CM and found that PFN2 expression in the CM was significantly decreased (Figure 5E), which indicated that PFN2 was mainly loaded by the exosomes. When cultured with exosomes depleted H446 CM, the migration and tube length were attenuated in HUVEC cells (Figure 5F, 5G). These results demonstrated that PFN2 is the critical factor contributing to HUVEC cells angiogenesis via exosomes. Then, we investigated whether the exosome secreted from H446 could be internalized by ECs. The results showed that H446-exo was taken up by ECs in a time-dependent manner and reached a maximum level after 6 h (Figure 5H). Thus, we examined the effect of H446-OE-exo and H446-KD-exo on HUVEC cell biological function. As expected, the migration and tube lengths of HUVEC cells were significantly higher in the H446-OE-exo group (Figure 5I, 5J) and lower in the H446-KD-exo group (Figure 5K, 5L) compared to those in their corresponding controls. Our data indicate that exosomes from SCLC cells overexpressing PFN2 enhance the angiogenesis ability of ECs.

### SCLC cells internalized exosomes more rapidly but with no significant specificity

**Figure 6:**
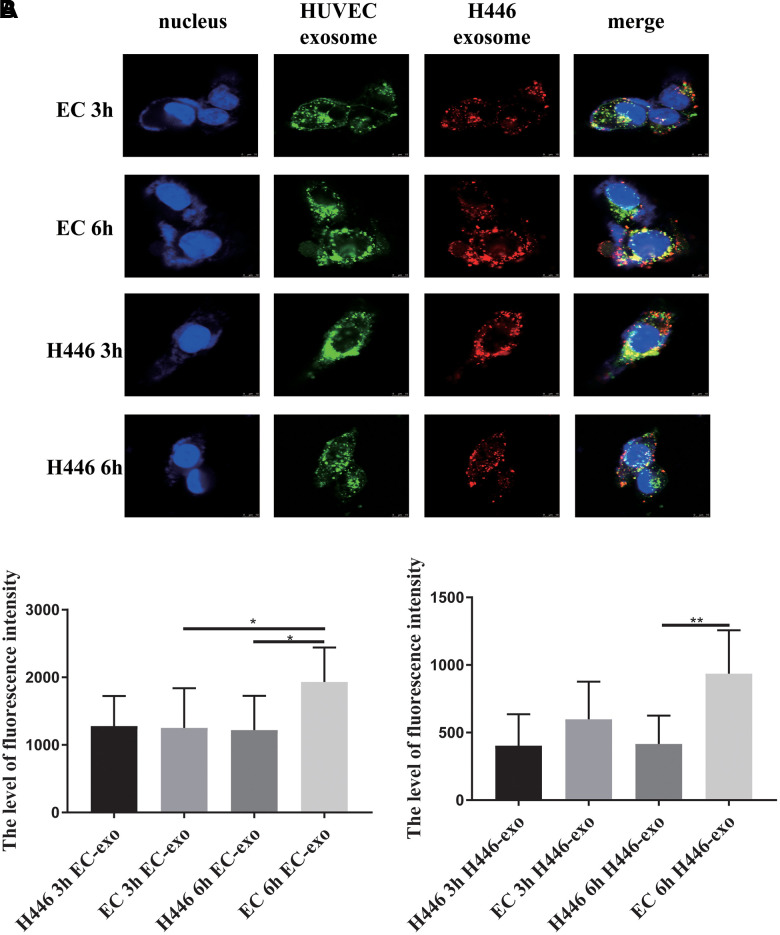
**Dynamic and comparison of exosomes (derived from H446 or HUVEC cells) internalized by H446 or HUVEC cells.** H446 and HUVEC cells could internalize exosomes from H446 and HUVEC cells at 3 h and 6 h (**A**). The amount of exosomes derived from H446 or HUVEC cells internalized by H446 or HUVEC cells was measured. H446 and HUVEC cells internalized the similar amount of exosomes at 3 h. However, at the 6 h, HUVEC cells internalized more exosomes than H446 cells (**B**).

To deeply understand exosome mediated cross-talk between ECs and SCLC cells, we explored whether there were differences in SCLC cells or ECs as recipient cells to internalize exosomes from different origins of cells and whether there was a specificity or superiority when the SCLC cells and ECs internalized exosomes derived from themselves. We labeled exosomes derived from SCLC cells with PKH26 (red) and those derived from ECs with PKH67 (green) and mixed them in equal proportion. Next, SCLC cells and ECs were treated with mixed-labeled exosomes. In this experiment, we found that H446 could internalize exosomes from H446 and ECs, and the amount of exosomes internalized by H446 at 2.5 h was similar to that at 3 h. In contrast, ECs could internalize more exosomes at 3 h than at 2.5 h (Supplementary Figure 2). After co-culturing for 3 h or 6 h, we detected the amount of exosomes internalized in SCLC cells and ECs, respectively. As shown in Figure 6A, 6B, both H446 and HUVEC cells demonstrated similar uptake of exosomes after 3 h of incubation. However, at the 6 h test end time-point, H446 cells had lesser intake of both H446-exo and EC-exo than ECs. These results indicated that although there was no significant specificity for SCLC cells and ECs when they internalized exosomes, ECs could internalize more exosomes than SCLC cells.

### PFN2 influences SCLC cells and ECs via different pathways

**Figure 7:**
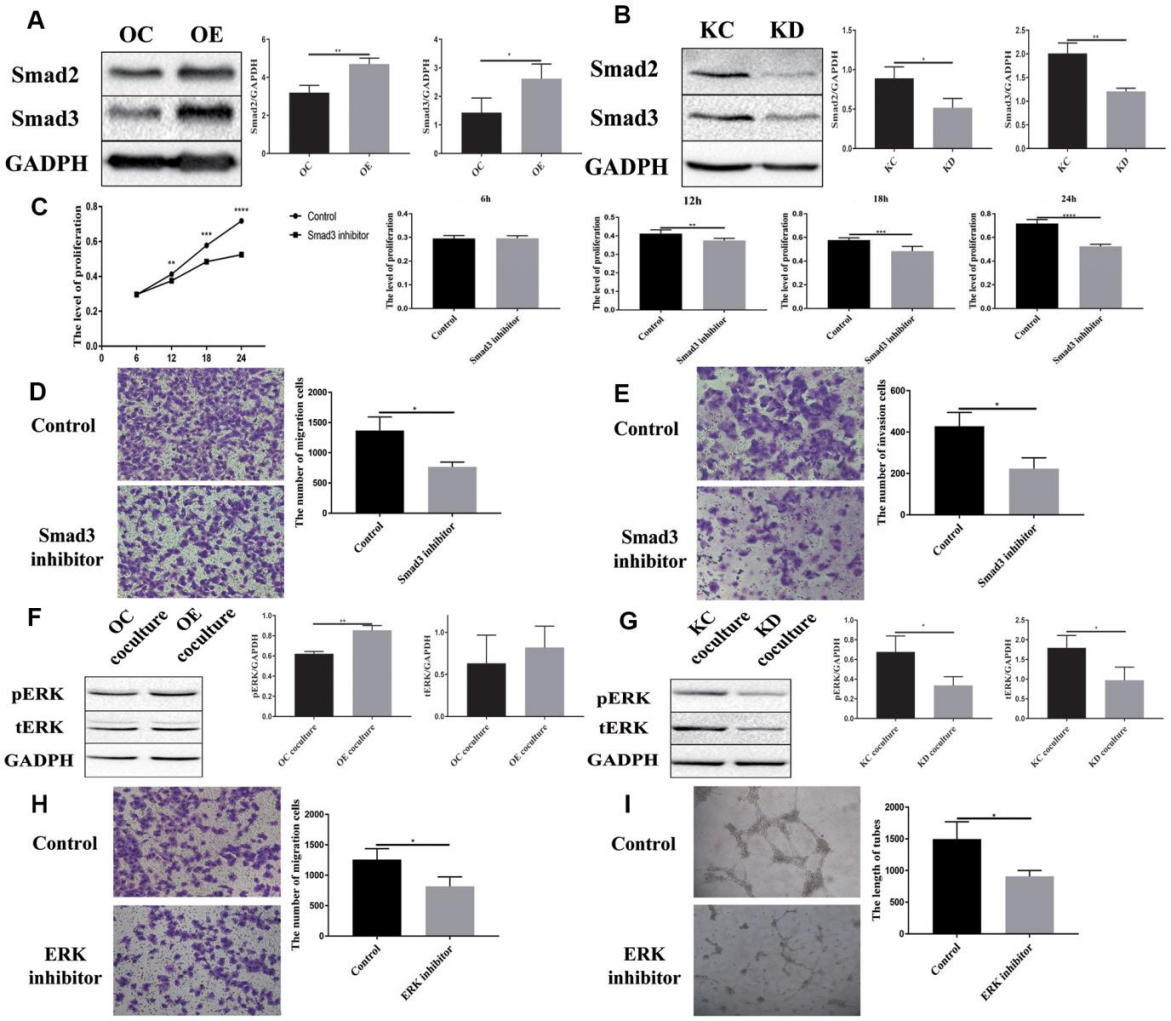
**Downstream signaling molecules activated by PFN2 are Smad2/3 in H446 cells and pERK in HUVEC cells.** Smad2 and Smad3 expression is increased in H446-OE (**A**) but decreased in H446-KD (**B**). Smad3 inhibitor could impede the function of PFN2 in promoting the proliferation (*P*=0.9279, 0.0030, 0.0004, and 0.000 at 6 h, 12 h, 18 h, and 24 h, respectively) (**C**), migration (*P*=0.0123) (**D**), and invasion (*P*=0.0136) (**E**) of H446 cells. pERK and tERK expression in HUVEC cells co-cultured with H446-OE-exo is upregulated (**F**), whereas the expression in those co-cultured with H446-KD-exo is downregulated (**G**). ERK inhibitor could impede the function of PFN2 in promoting the migration (*P*=0.0333) (**H**) and tube formation ability (*P*=0.0238) of ECs (**I**).

A previous study has shown that PFN2 promotes the growth and metastasis of lung cancer by epigenetic regulation of Smad2 and Smad3 [16]. Thus, we tested Smad2 and Smad3 expression in H446-OE and H446-KD cell lines. Similar to the findings in NSCLC cell line, PFN2 OE increased Smad2 and Smad3 expression, whereas PFN2 knockdown inhibited the expression (Figure 7A, 7B). More importantly, the Smad3 inhibitor ((E)-SIS3) could impede the function of PFN2 in promoting the proliferation, migration, and invasion abilities of H446-OE (Figure 7C–7E). We propose that PFN2 may promote the growth and metastasis of SCLC by upregulating Smad2 and Smad3 expression.

Previous studies revealed that the MAPK/ERK pathway plays a crucial role in VEGF-mediated angiogenesis [22, 23]. We detected ERK phosphorylation in ECs co-cultured with SCLC cells. After co-culturing with H446-OE, ERK phosphorylation (pERK) in ECs was remarkably increased compared to that in cells co-cultured with H446-OC, whereas after co-culturing with H446-KD, pERK in ECs decreased compared to that in cells co-cultured with H446-KC (Figure 7F, 7G). However, PI3K, AKT, p-AKT, Smad2, and Smad3 expression exhibited no difference among ECs co-cultured with OE-H446, OC-H446, KD-H446, and KC-H446 (Supplementary Figure 3). We also found that an ERK inhibitor (SCH772984) could impede the function of PFN2 in promoting the migration and tube formation ability of ECs (Figure 7H, 7I). These data indicate that PFN2 enhances EC angiogenesis through the MAPK/ERK pathway.

### PFN2 promoted the growth, metastasis, and angiogenesis of SCLC *in vivo*

**Figure 8:**
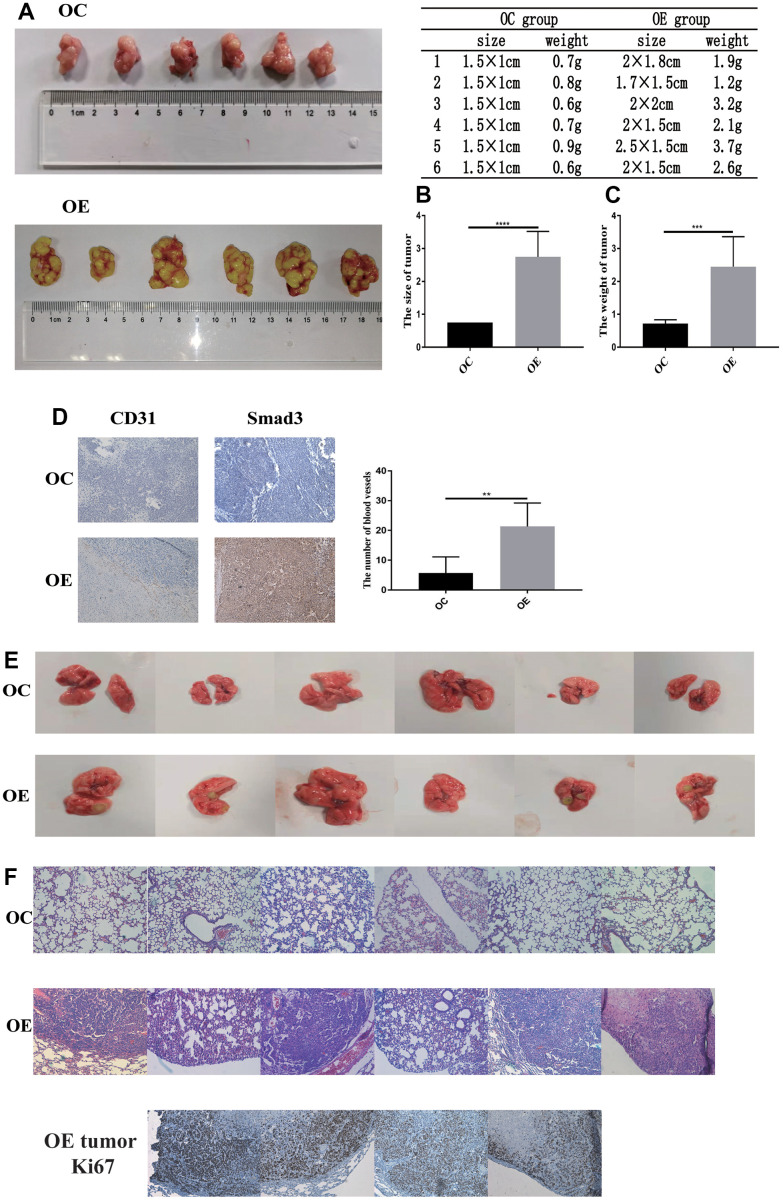
**PFN2 promotes tumor growth and metastasis *in-vivo*.** The size and weight of tumor in the H446-OE group is significantly higher than those of tumor in the H446-OC group in xenograft mice model (**A–C**). There is a significantly higher number of blood vessels in tumor in the H446-OE group than in that in the H446-OC group; meanwhile, Smad3 expression is higher in the H446-OE group than in H446-OC group (**D**). H446-OE cells could remarkably promote metastasis *in vivo* (**E**), confirmed by hematoxylin and eosin staining (**F**); the number of Ki67 positive cell in metastatic tumor in the lung is significantly higher than that in the adjacent tissues (**F**).

To further validate the function of PFN2 in SCLC *in vivo*, CB17-SCID mice were subcutaneously injected with H446-OE or H446-OC. Both size and weight of tumors in SCID mice were significantly higher in the H446-OE group than in the H446-OC group (Figure 8A–8C). Consequently, we detected CD31 expression to evaluate the number of blood vessels in the tumors. The results revealed that the tumors of the H446-OE group harbored significantly more blood vessels than those of the H446-OC group (Figure 8D). Smad3 expression was higher in the H446-OE group than in the H446-OC group (Figure 8D). These results showed that PFN2 promoted the tumor growth of SCLC by upregulating Smad3 and increasing angiogenesis in the tumors.

To investigate the function of PFN2 in the metastasis of SCLC, CB17-SCID mice were injected with H446-OE or H446-OC through tail veins. As shown in Figure 8E, no mice (0/6, 0%) harbored detective metastasis in the OC group, whereas four mice (4/6, 66.7%) displayed obvious tumor metastasis in the OE group. We confirmed the metastasis by pathological analysis using HE staining (Figure 8F). These results revealed that PFN2 promoted the metastasis of SCLC. Cell proliferation in tumors metastasized in the lung was remarkably higher than adjacent normal tissues in the H446-OE group (Figure 8F).

## DISCUSSION

SCLC is a highly lethal cancer with features that are very different from those of other lung cancers in its pathology, molecular, biological, and clinical characteristics [24]. However, due to its propensity to metastasize and rapid relapse to chemotherapy, there have been no significant therapeutic advances over the past decades, and it is considered as a recalcitrant cancer [24]. Thus, comprehensive molecular analyses are urgently needed to improve clinical treatment strategies by identifying potential new therapeutic targets. In the present study, we reported, for the first time, that PFN2, a cytoskeletal regulator, plays an important role in the metastasis and growth of SCLC. PFN2 OE or enriched presence of PFN2 in exosomes significantly promoted SCLC tumor cell growth *in vitro* and *in vivo*; PFN2 also enhanced tumor angiogenesis through exosomes. These effects observed in both SCLC cells and ECs may have been exerted through Smad2/3 and pERK pathways, respectively. Interestingly, when SCID mice xenografted with PFN2-overexpressing SCLC cells were treated with exosomes originating from PFN2-knockdown SCLC cells, their tumors were smaller than those in the untreated mice (Supplementary Figure 4), implying that PFN2 may be a valuable therapeutic target for SCLC in clinical practice. Because cancer cell migration and invasion are prerequisites for metastasis, PFN2 OE significantly promoted proliferation, migration, and invasion, demonstrating a novel mechanism underlying PFN2 mediated tumor metastasis [25].

Hypervascularity is an important characteristic of SCLC [24]. However, investigations on SCLC angiogenesis or molecular mechanisms underlying tumor angiogenesis are relatively limited [26]. A recent report emphasized that decreased FMOD expression inhibited tumor angiogenesis of SCLC by downregulating the angiogenic factors of ECs [27]. In SCLC studies, some traditional vascularity or angiogenesis genes, such as VEGFA, HIF-α, and bFGF, and Notch signaling pathways have been investigated and applied as targeted therapy [28]. However, obvious advances have not been made by targeting these genes. Targeting VEGF and VEGFA in SCLC has only shown moderate therapeutic improvements [28]. In addition, VEGF is also a key mediator of angiogenesis in healthy tissues, with a general side effect on patients being another crucial concern. The function of PFN2 has been reported in head and neck cancers [11, 12], ovarian cancer [29], and NSCLC [16]. Our present study showed that PFN2 promoted the progression of SCLC *in vitro* and *in vivo*. Furthermore, we revealed that PFN2 regulated tumor angiogenesis in the tumor microenvironment through cancer-derived exosomes. PFN2 expression is low in most normal tissues or organs, except for the neuronal system [30]. Thus, PFN2 downregulation will have a therapeutic effect on SCLC with minimal side effects on healthy tissues. Our studies indicated that PFN2 promoted SCLC development by enhancing tumor angiogenesis.

Exosomes, which are membrane microvesicles secreted by cells, are important carriers of various bioactive molecules and are well-known promoters of the invasion and migration of cancer cells [25–31]. An increasing number of studies have revealed that cancer-derived exosomes could influence cancer growth, progression, and metastasis in a lymphatic and vascular manner [32, 33]. The most common understanding of exosome function focuses on the communication or cross-talk of inter-cells, including inter-cancer cells, cancer-stromal cells, cancer-vascular cells, and cancer-lymphatic cells [33–35]. In the present study, exosomes derived from SCLC cells interacted with SCLC cells and ECs directly; PFN2 promoted the growth and metastasis of SCLC. Most importantly, our work demonstrated that the uptake of exosomes in either SCLC cells or ECs was not significantly different at 3 h; however, EC intakes more either H446-exo or EC-exo at 6 h of the test end time-point, suggesting that SCLC cells could effectively communicate with EC through exosomes, although ECs uptake more exosomes than SCLC cells, similar to a previous report showing that uptake of exosomes exhibited cell-origination specificity and efficiency [36]. Our work, for the first time, showed that exosomal PFN2 plays a role in communication between SCLC cells and ECs.

Our study has several limitations. First, our study on PFN2 was based on small SCLC sample sizes with incomplete clinical information collected, which may have limited our analysis of the relationship between PFN2 expression level and metastasis, overall survival, or other clinical features. Second, as an intracellular protein, PFN2 may be initially released from cells by exosomes; downregulating PFN2 as a therapeutic strategy would not be easy. We injected KD-exo into xenografted mice with OE-SCLC cells and found a smaller tumor bulk. Nevertheless, it is a preliminary study and KD-exo resulted in the inhibition of tumor growth. Finally, we focused solely on the migration, proliferation, and invasion of SCLC cells and did not investigate the role of PFN2 in cell apoptosis, autophagy, adherence, or inflammation. We only validated the molecular mechanism reported by Tan et al. [16].

## MATERIALS AND METHODS

### Clinical specimens

The clinical specimens were collected from Beijing Chest Hospital from March 2018 to September 2019. A total of 95 SCLC samples were obtained from patients diagnosed with SCLC. Normal lung tissues were collected from 6 corresponding adjacent normal tissues of SCLC patients and 10 healthy individuals as controls. This study was approved by the Institutional Research Ethics Committee of the Capital Medical University (Permit No. Z2019SY031). Informed consent was obtained from each patient before sample collection.

### Cell cultures

The SCLC cell line H446 and endothelial HUVEC cells were purchased from the Shanghai Institute of Cell Biology of the Chinese Academy of Sciences (Shanghai, China). H446 and HUVEC were cultured in RPMI 1640 medium (Corning) supplemented with 10% fetal bovine serum (FBS) (Vistech) in a humidified incubator at 37° C with 5% CO_2_.

### Cell transfection

H446 cells overexpressing PFN2 and the corresponding control cells (OC) were established using the pLenti-GIII-CMV-GFP-2A-Puro vector (ABM). H446 cells knockdown PFN2 (KD) and the corresponding control cells (KC) were established using the piLenti-siRNA-GFP vector (ABM). Cells were transduced for 24 h with recombinant lentivirus and cultured for 72 h. The transduction efficacy was verified by GFP expression and detected using western blot.

### Immunohistochemistry

For IHC staining, the tissues were fixed, embedded, and cut into sections, followed by deparaffinization in xylene and ethanol and rehydration in water. Antigen retrieval was performed by heating the slides in a microwave for 20 min in sodium citrate buffer (pH 6.0) and quenching in hydrogen peroxide (3%) to block endogenous peroxidase activity and then washing with PBS. The slides were incubated with primary antibodies against PFN2 (Abcam), Smad3 (Immunoway), and CD31 (Abcam) at 4° C overnight. Horseradish peroxidase (HRP)-conjugated goat anti-mouse antibody (Zsgb-Bio) or HRP-conjugated goat anti-rabbit antibody (Zsgb-Bio) was used as the secondary antibody.

### Cell co-culture assay

The conditioned medium of OE, OC, KD, or KC H446 cells was used to co-culture EC for 24 h. For co-culture with cells, OE, OC, KD, or KC H446 cells were seeded in the upper chamber of a co-culture system equipped with a 0.4-μm pore membrane, and HUVEC cells (3×10^5^/well) were placed in the lower chamber for 24 h. All cells were incubated in RPMI 1640 medium with 10% exosome-free FBS.

### Cell proliferation assay

For MTT cell viability assay, the cells (H446 or HUVECs) were seeded in 96-well plates (Nunc) for 24 h and MTT reagent (5mg/mL) was added to each well. After a 4-h incubation, the media/MTT was aspirated and 100 μL DMSO was added to each well. The plate was incubated for 20 min, and the absorbance was measured in single-wavelength mode (490 nm) using the SpectraMax M5 multi-mode microplate reader (Molecular Devices). The results were normalized to the medium alone control.

### Transwell migration and invasion assays

For cell migration assay, the cells were seeded in serum-free RPMI 1640 in the upper chamber. The lower chamber was filled with RPMI 1640 containing 10% FBS. After 12 h, the upper chamber was washed with PBS, scraped on the side of the filter with a cotton swab, and fixed with 4% paraformaldehyde (PFA) for 5 min. Cells adherent to the bottom were stained with hematoxylin for 10 min. The positively stained cells were imaged and examined under a Leica DMI4000B microscope (Leica).

For cell invasion assay, the cells were plated onto the upper chamber precoated with a basement membrane matrix (Corning) filled with serum-free medium, while the bottom chamber was filled with RPMI 1640 supplemented with 10% FBS. Cells were incubated for 16 h. After washing with PBS, the cells in the upper chamber were scraped on the side of the filter with a cotton swab and fixed with 4% PFA for 5 min. Cells adherent to the bottom were stained with hematoxylin for 10 min and then washed 3 times with PBS. The positively stained cells on the underside of the filters were imaged and examined under a Leica DMI4000B microscope (Leica).

### Tube formation assay

HUVEC cells were diluted with serum-free RPMI 1640 and 2×10^4^ cells/well in 100 μl were added to a 96-well culture plate precoated with basement membrane matrix (Corning). The plate was incubated at 37° C for 12 h. Tube formation was visualized under an inverted microscope. The tube structures were randomly selected from three different fields and imaged under a Leica DMI4000B microscope (Leica).

### Exosome isolation from the culture medium

H446 or HUVEC cells were cultured with exosome-free FBS media for 48 h, the supernatant was collected, and the exosomes were enriched by ultracentrifugation. The final exosome pellet was resuspended in PBS and validated using western blot, electron microscopy (TEM), and nanoparticle tracking analysis (NTA). The exosome pellet was resuspended in PBS, stored at -80° C, and thawed prior to use. The protein concentration of the exosome fraction was measured using a BCA protein quantitation kit (ThermoFisher).

### Exosome internalization and exosome-co-culture assay

For exosome internalized experiments, the exosomes were labeled with PKH26 (red) or PKH67 (green) (Sigma) following the manufacturer’s protocol. A total of 10 μg of exosomes was resuspended in 100 μl PBS and added into 1×10^4^ H446 cells or HUVEC cells to incubate for 3 or 6 h. The cells were then washed with PBS 3 times and fixed with 4% PFA for 5 min, followed by washing one more time with PBS. The cells were then treated with Hoechst for 5 min and washed to add the Antifade Mounting Solution. The localization of exosomes in cells was examined under a TCS SP5 confocal microtelescope (Leica).

For the exosome co-culture assay, 50 μg exosomes were added to 7 mL RPMI 1640 supplemented with 10% FBS. The culture medium was filtered through a 0.45-μm cell strainer. The H446 cells or HUVECs were treated with the culture medium for 24 h.

### Electron microscopy

Briefly, the exosomes were fixed in 2% PFA and absorbed by a Formvar–carbon-coated 400 mesh copper grid (Electron Microscopy Sciences) for 20 min. The grid was then fixed with 1% glutaraldehyde for 5 min and stained with uranyl oxalate solution at pH 7 for 5 min and then treated with a 9:1 ratio of 2% methyl cellulose at pH 4 and 4% uranyl acetate for 10 min. Finally, the air-dried grids were imaged under a JEM-2100 electron microscope at 80 kV.

### Western blot analysis

Protein samples were prepared using the BCA Protein Assay Kit (Thermal Fisher). A total of 50 μg of protein was separated on 10% SDS-PAGE and subsequently transferred onto polyvinylidene difluoride membranes. The membranes were blocked with 5% bovine serum albumin (BSA) for 1 h before being incubated overnight at 4° C with primary antibodies against PFN2 (Abcam), CD31 (Abcam), TSG101 (Abcam), Alix (Proteintech), Smad2 (Huabio), Smad3 (Immunoway), AKT (CST), p-AKT (CST), ERK (CST), p-ERK (CST), PI3K (CST), or GAPDH (CST). The membranes were washed three times with PBST and then incubated with HRP-conjugated secondary antibodies (Yeasen) for 1 h. After the final wash with TBST, the proteins were detected using enhanced chemiluminescence reagents.

### Enzyme-linked immunosorbent assay

The enzyme-linked immunosorbent assay (ELISA) was performed according to the experimental protocol. Briefly, different concentrations of standards were prepared and the samples were diluted. The standards and samples were added to the test plate. The plate was then incubated at 37° C for 30 min and washed 5 times. The peroxidase reagent was added, and the plates were incubated at 37° C for 30 min. The plates were then washed 5 times, and the reagents A and B were added. The plates were incubated at 37° C for 10 min, the stop reagent was added, and the absorbance values were detected at 490 nm. A standard curve was drawn based on the results of standards measurements, and the concentration of the samples was determined using the absorbance values of the samples.

### Animal experiments

For the tumorigenicity assay, a total of 12 male SCID mice (aged 5 weeks old) purchased from Charles River, Beijing, were randomly grouped into two groups (*n* = 6 in each group). Subsequently, the SCID mice were subcutaneously injected with the H446 cells overexpressing PFN2 (5×10^7^ cells/200 μL) and its respective control. After 4 weeks, the mice were euthanized, and the size and weight of the resected implanted tumors were measured. The tumor volume was calculated as V = W^2^×L×0.5. The subcutaneous tumors were fixed in 4% neutral formaldehyde overnight. After embedding in paraffin, the samples were cut into sections with 5-μm thickness to perform IHC for CD31 and Smad3.

For the tumor metastasis assay, the mice were injected with the H446 cells overexpressing PFN2 and its control (2×10^6^ cells/200 μL) via the tail vein. After 3 months, the mice were sacrificed, and the presence of metastatic tumors were examined in the liver and lungs. HE and Ki67 staining were then performed. Animal experiments were approved by the Scientific and Ethics Committee of Capital Medical University (Permit No. AEEI-2018-154).

### Statistics

Statistical analysis was performed using GraphPad Prism 7 (GraphPad Software). Unless otherwise indicated, data were analyzed using two-tailed Student’s *t*-test for two groups and one-way ANOVA followed by the Newman–Keuls multiple comparisons test was used to analyze statistical significance among multiple groups. Data are shown as mean ± SEM. A *P*-value (<0.05) was considered statistically significant.

## Supplementary Material

Supplementary Figures
