# Supplementary material for: Profilin 2 promotes growth, metastasis, and angiogenesis of small cell lung cancer through cancer-derived exosomes
Source: Aging (Albany NY). 2020 Nov 21;12(24):25981–99. doi: 10.18632/aging.202213 (PMC7803489; doi:10.18632/aging.202213)
Supplement: Supplementary Figures [file aging-12-202213-s001.pdf]

SUPPLEMENTARY FIGURES

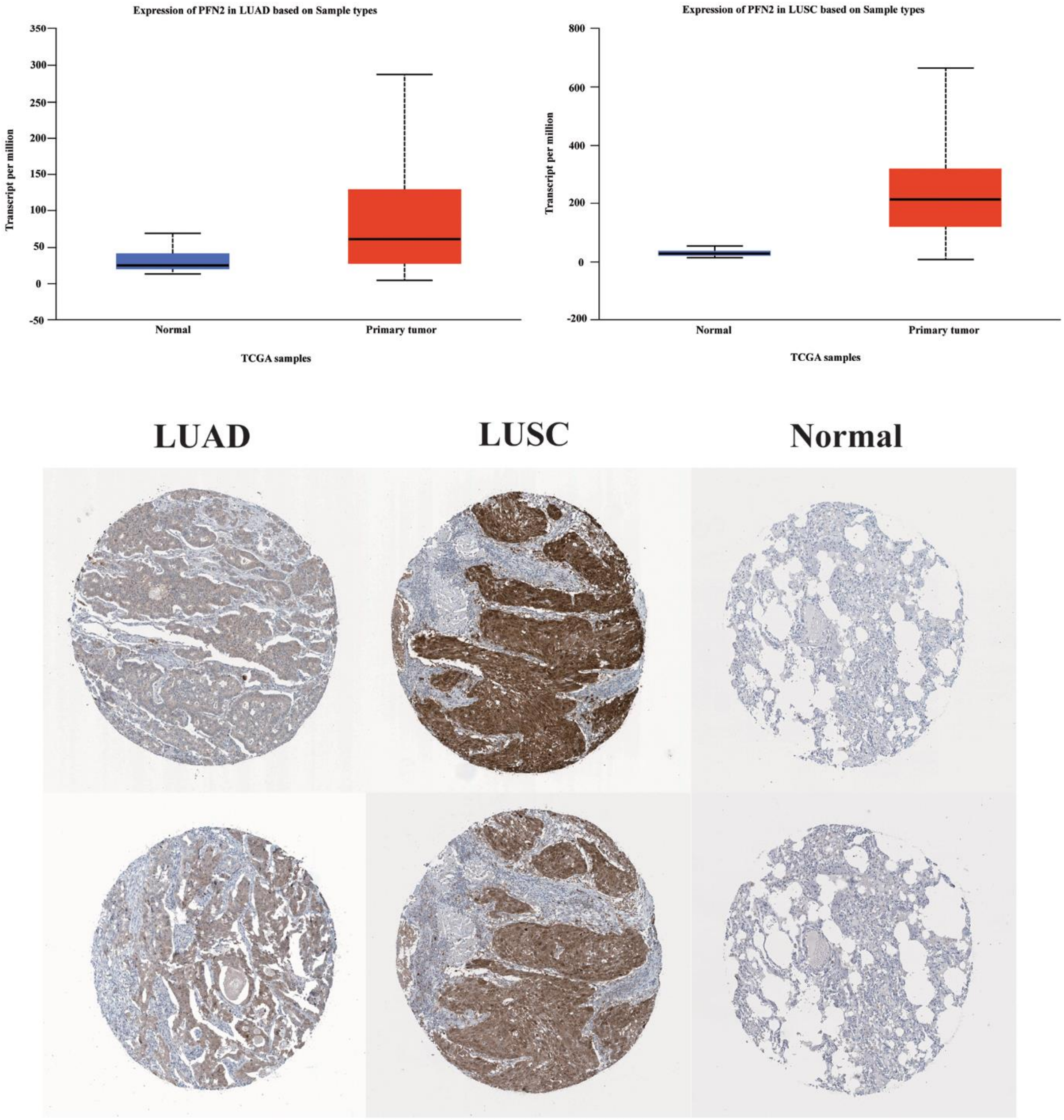

Supplementary Figure 1. PFN2 is highly expressed in non–small-cell lung cancer (NSCLC) but not in normal lung tissues searched in the TCGA database.

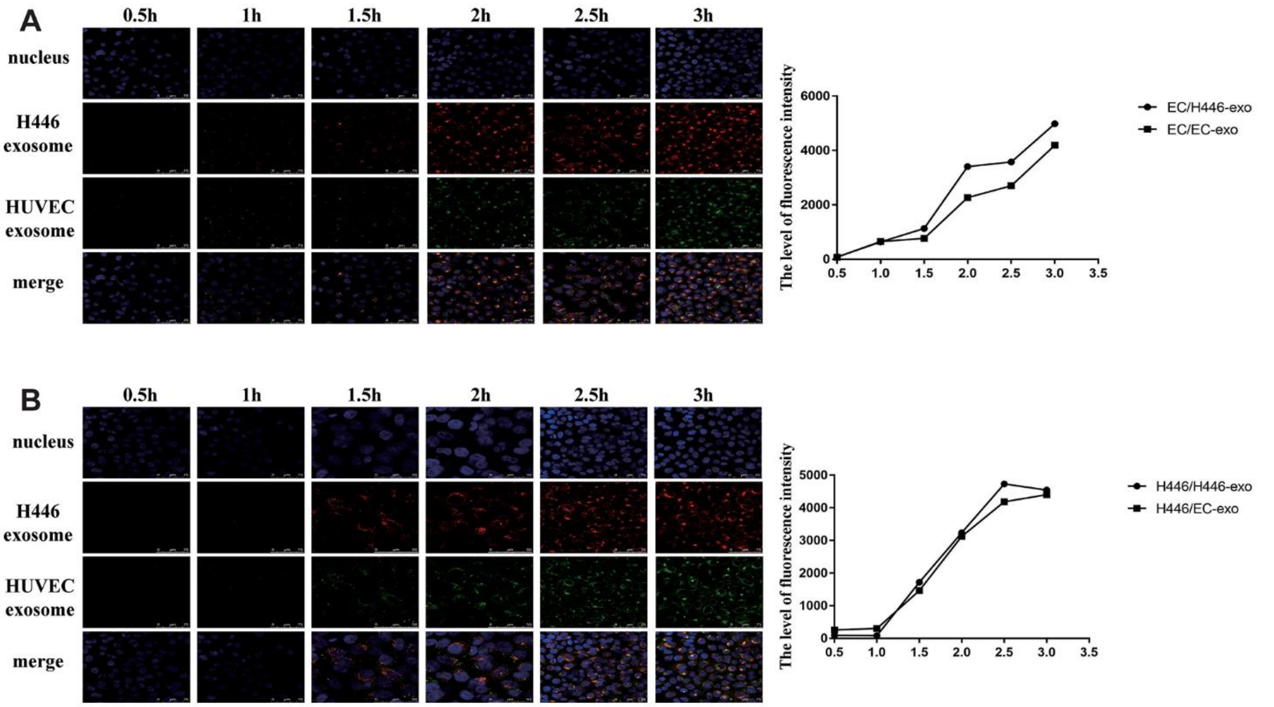

Supplementary Figure 2. The number of exosomes internalized by H446 cells and ECs within 3 h. H446 could internalize exosomes derived from H446 cells and ECs, and the amount of exosomes internalized by H446 cells at 2.5 h was similar with that internalized at 3 h (**A**). Different from H446 cells, ECs could internalize more exosomes at 3 h than at 2.5 h (**B**). EC, endothelial cells.

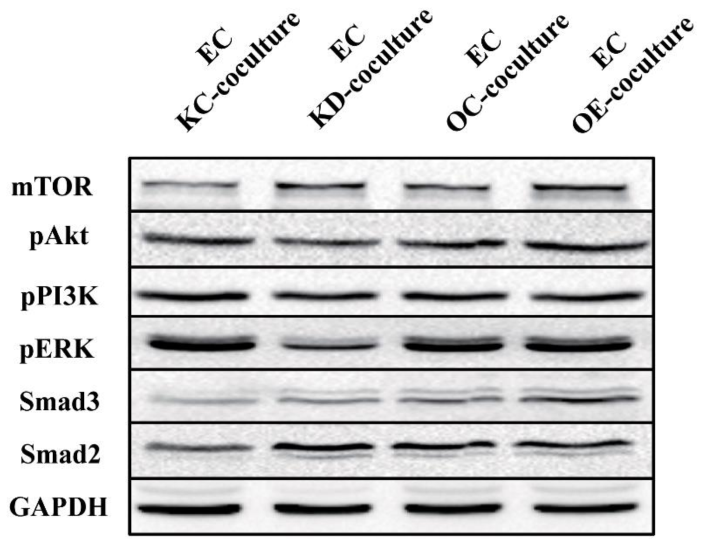

Supplementary Figure 3. PI3K, AKT, p-AKT, Smad2, and Smad3 expression exhibits no difference between H446-OE and H446-OC co-cultured ECs.

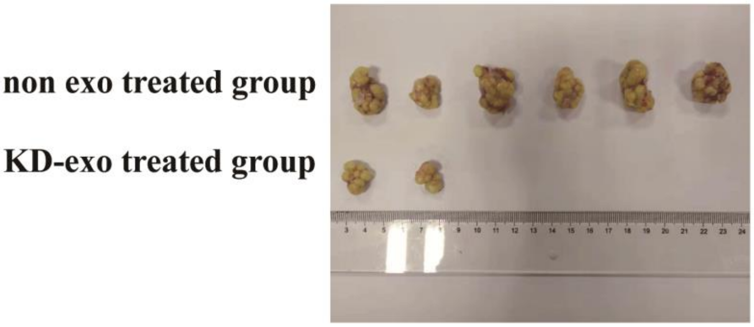

	KD-exo treated group		non exo treated group(OE group)	
	size	weight	size	weight
1	1.5×1cm	0.9g	2×1.8cm	1.9g
2	1.3×1cm	0.8g	1.7×1.5cm	1.2g
3			2×2cm	3.2g
4			2×1.5cm	2.1g
5			2.5×1.5cm	3.7g
6			2×1.5cm	2.6g

Supplementary Figure 4. Xenograft tumor mice model with PFN2-overexpressing SCLC cells have smaller tumor size in the group treated by exosomes derived from PFN2-knockdown SCLC cells than in the non-treated group.
